# A novel feature for monitoring the enzymatic harvesting process of adherent cell cultures based on lens-free imaging

**DOI:** 10.1038/s41598-022-22561-x

**Published:** 2022-12-23

**Authors:** T. Deckers, J.-M. Aerts, V. Bloemen

**Affiliations:** 1 M3-BIORES, KU Leuven, Leuven, Belgium; 2 Surface and Interface Engineered Materials (SIEM), Campus Group T, KU Leuven, Leuven, Belgium; 3 Prometheus, Division of Skeletal Tissue Engineering Leuven, KU Leuven, Leuven, Belgium

**Keywords:** Biomedical engineering, Software, Tissue engineering, Mesenchymal stem cells, Image processing, Time-lapse imaging

## Abstract

Adherent cell cultures are often dissociated from their culture vessel (and each other) through enzymatic harvesting, where the detachment response is monitored by an operator. However, this approach is lacking standardisation and reproducibility, and prolonged exposure or too high concentrations can affect the cell’s viability and differentiation potential. Quantitative monitoring systems are required to characterise the cell detachment response and objectively determine the optimal time-point to inhibit the enzymatic reaction. State-of-the-art methodologies rely on bulky imaging systems and/or features (e.g. circularity) that lack robustness. In this study, lens-free imaging (LFI) technology was used to develop a novel cell detachment feature. Seven different donors were cultured and subsequently harvested with a (diluted) enzymatic harvesting solution after 3, 5 and 7 days of culture. Cell detachment was captured with the LFI set-up over a period of 20 min (every 20 s) and by optimising the reconstruction of the LFI intensity images, a new feature could be identified. Bright regions in the intensity image were identified as detaching cells and using image analysis, a method was developed to automatically extract this feature, defined as the percentage of detached cell regions. Next, the method was quantitatively and qualitatively validated on a diverse set of images. Average absolute error values of 1.49%, 1.34% and 1.97% were obtained for medium to high density and overconfluent cultures, respectively. The detachment response was quantified for all conditions and the optimal time for enzyme inhibition was reached when approximately 92.5% of the cells were detached. On average, inhibition times of 9.6–11.1 and 16.2–17.2 min were obtained for medium to high density and overconfluent cultures, respectively. In general, overconfluent cultures detached much slower, while their detachment rate was also decreased by the diluted harvesting solution. Moreover, several donors exhibited similar trends in cell detachment behaviour, with two clear outliers. Using the novel feature, measurements can be performed with an increased robustness, while the compact LFI design could pave the way for in situ monitoring in a variety of culture vessels, including bioreactors.

## Introduction

For autologous cell and tissue engineering therapies, cells are isolated from the patient, expanded in vitro until the required number of cells is obtained and implanted in vivo at the defect site^1^. The expansion phase of the cells is taking place either in suspension or while adhered to a culture surface. Most cell types are anchorage-dependent and are therefore grown as monolayers on artificial substrates^2^. To promote further cell growth and thereby scale towards clinically relevant cell numbers, the cells are periodically passaged at confluency. Enzymatic (e.g. trypsin, TrypLE and accutase) and non-enzymatic [scraping, cell dissociation buffer (CDB) and ethylenediamine tetra-acetic (EDTA)] methods^3^ exist to dissociate cells from their substrate. Mechanical harvesting such as cell scraping is a labour-intensive process and can result in irreversible cell damage. Moreover, it is not applicable for closed and multi-layered vessels. Therefore, a viable alternative is enzymatic harvesting, where proteolytic enzymes hydrolyse specific peptide bonds that enable the cells to adhere to the vessel^4^. However, prolonged exposure to trypsin or other proteolytic enzymes as well as an exposure to too high concentrations of these enzymes can cause adverse cell effects such as genomic instability and reduced differentiation efficiency^5–9^ and eventually result in irreversible cell damage. Monitoring the enzymatic harvesting process is therefore a crucial step to meet the cell quality requirements within the field and obtain reproducible cell batches.

In a lab environment, it is standard practice to manually inspect the detachment of adherent cells. An operator monitors the ‘floating’ or circularity of the cell population at regular times with the microscope^10^, to decide when to stop the enzymatic reaction. Cells undergo specific morphological changes upon detachment, such as an increased circularity/roundness and intensity, which can form the basis for automated monitoring technologies. In Viazzi et al*.*^11^, bright-field microscopy was used to automatically monitor the cell circularity during cell harvesting and predict the enzyme inhibition time. However, cell circularity as a feature requires the accurate segmentation of individual cells. Although advanced models have been developed to achieve reasonable performance for conventional microscopy^12–16^, they are often tailored to specific cell types and lack robustness for high cell densities. Moreover, these microscopy set-ups are often bulky and/or expensive and therefore lack scalability. Lens-free microscopes, on the other hand, offer several advantages including a large field-of-view, compact and cost-effective design and potential for integration with other components^17^. In Kesavan et al*.*^18^*,* lens-free imaging was used to monitor the attachment or detachment (upon cell death) response of cells using template matching or cells’ circularity. Here, similar concerns about the segmentation of single cells are raised, while LFI often does not provide a reliable morphological representation of the cells. In general, it is also a question how well cell circularity correlates to cell detachment across different cell types (e.g. round-shaped cells) and densities.

In this study, LFI technology was used to develop a novel feature for monitoring cell detachment, eliminating the shortcomings associated with the circularity feature while exploiting the advantages of lens-free imaging. It was hypothesized that this feature could better represent detaching cells while being more suitable for automated extraction across different cell types and cell densities. In order to verify this, the following objectives were defined: (i) identify a novel, image-based feature to quantify cell detachment and compare it to the gold standard (i.e. cell circularity/intensity). (ii) develop a method to automatically extract the feature. (iii) evaluate the detachment response of cells to the harvesting solution for different donors and conditions using the new feature. (iv) develop an approach to automatically determine the optimal time-point for inhibition of the enzymatic reaction.

## Materials and methods

### LFI set-up

**Figure 1:**
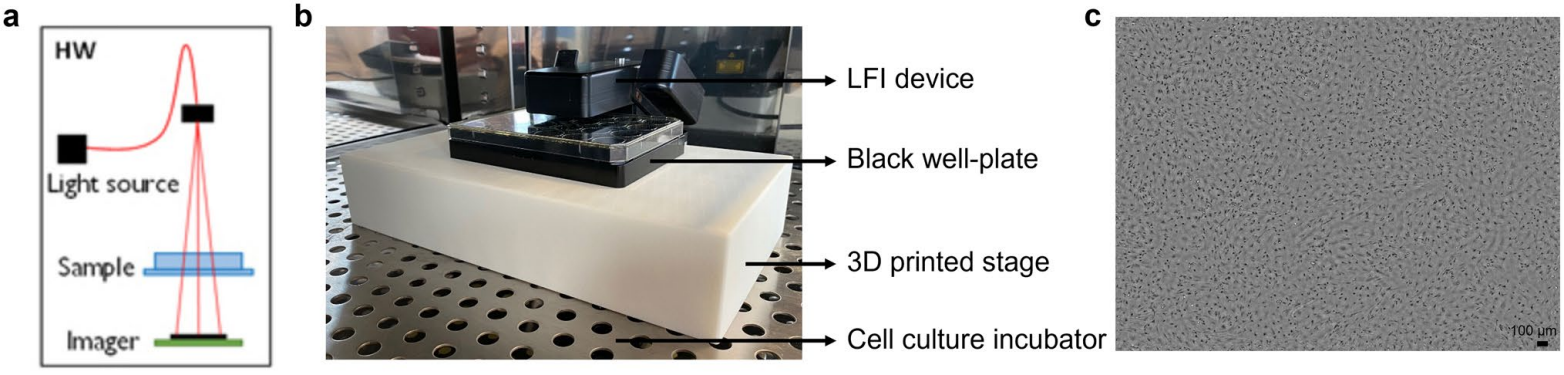
Lens-free imaging. **(a)** Schematic of the lens-free imaging set-up (figure adapted from ^31^). **(b)** Picture of the LFI microscope, mounted inside a standard cell culture incubator with a 3D printed stage for well-plate support. **(c)** Reconstructed phase image of detaching cells, acquired with the LFI set-up. The black scale bar represents 100 µm.

A lens-free imaging (LFI) device^19^, mounted inside a standard cell culture incubator (37 °C, 5% CO_2_ and 95% humidity, Thermo Fisher Scientific) with a 3D printed stage for 6-well plate support (Fig. 1), was used for continuous monitoring of the cell harvesting process. Since the acquisition speed could be a limiting factor due to heating of the image sensor, the temperature response was monitored for a high acquisition rate, as described in Supplementary Fig. S1.

### Cell expansion

Human periosteum-derived mesenchymal stem cells (hPDCs) were isolated from periosteal biopsies of seven different donors, as described by Roberts et al*.*^20^. Procedures were approved by the Ethical Committee for Human Medical Research (KU Leuven) and patient informed consent forms were obtained. The hPDC donors were expanded (5700 cells/cm^2^) at 37 °C, 5% CO_2_ and 95% relative humidity in high-glucose GlutaMAX™ Dulbecco’s modified Eagle medium (DMEM; Life Technologies, UK) containing 1 × 10^−3^ m sodium pyruvate and supplemented with 10% irradiated fetal bovine serum (HyClone FBS; Thermo Scientific, USA) and 1% antibiotic–antimycotic (100 units/ml penicillin, 100 mg/ml streptomycin, and 0.25 mg/ml amphotericin B; Invitrogen). Medium was changed every 3–4 days. At all passages, cells were harvested with TrypLE™ Express 1× (Life Technologies, UK). In this study, all experiments and methods involving these cells were performed in accordance with the relevant guidelines and regulations.

### Harvesting assay

A harvesting assay was performed involving different donors, cell densities and harvesting solutions. Seven different hPDC donors were seeded in 6-well plates (5700 cells/cm^2^, 5 wells per donor) in 3 ml of growth (DMEM-C) medium, which was completely refreshed after 3 days. In order to investigate the effect of culture time (related to cell density and matrix formation) and dilution of the enzymatic solution on the detachment rate of each donor, cells were cultured for 3, 5 and 7 days and enzymatically harvested with a standard (day 3, 5 and 7) and diluted (5 times in PBS, day 5 and 7) TrypLE Express solution. The following protocol was used for each well: (i) The growth medium was aspirated and the cells were washed 1× with phosphate buffered saline (PBS, 1 ml). (ii) The LFI was calibrated on the cells (in PBS) and a time-lapse experiment with an interval of 20 s was started. (iii) The PBS was aspirated and the ‘dry’ well was placed under the LFI. Reference images prior to the addition of TrypLE were taken. (iv) In between two acquisitions, 1 ml of TrypLE solution was added to the (open) well plate and the incubator was closed. The harvesting process was monitored for 20 min and all images were acquired without a well plate cover.

### Feature engineering and image reconstruction

LFI phase and intensity images, respectively equivalent to conventional phase-contrast and bright-field microscopy, were reconstructed^19^. Several reconstruction parameters, such as the reconstruction method [3L or iterative phase recovery (IPR)], the iteration count, the IPR threshold (80–120) and the focus level, were varied for the intensity image to identify a new feature for measuring cell detachment. Based on visual inspection, the following reconstruction parameters were chosen: temperature (37 °C), clipping (no), phase rotation (auto). More specifically, the phase image was reconstructed using the 3L method (iteration count: 5), and the intensity image with the IPR method (IPR threshold: 90, iteration count: 10). For each dataset, the reconstruction depth was determined manually on attached cells around the center of the image. The raw image was cropped with the following pixel parameters: [836:2236, 1198:2898].

### Image processing and feature extraction

**Figure 2:**
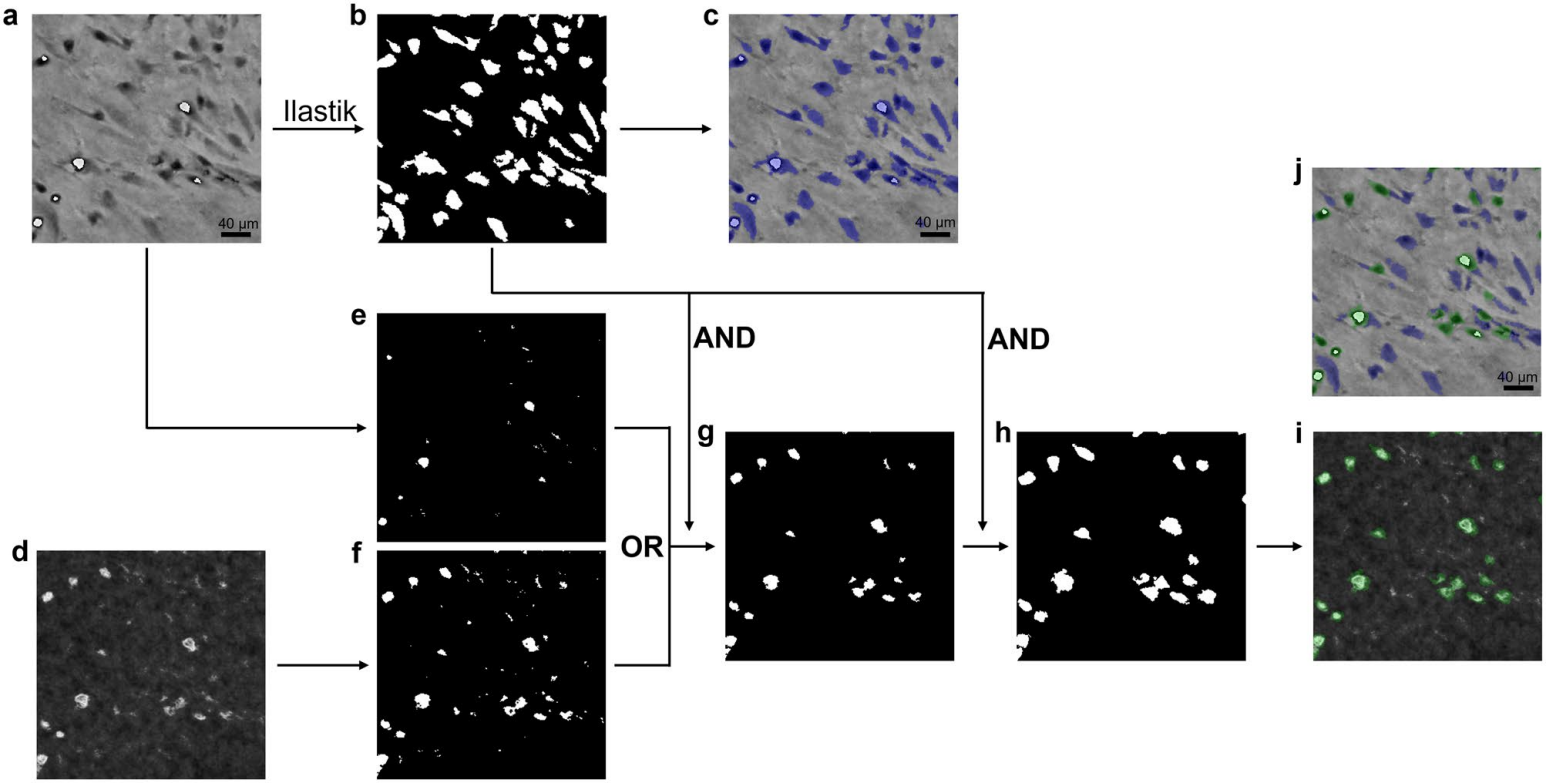
Automated feature extraction procedure. (**a**) Raw phase image. (**b**) The cell mask obtained with the Ilastik software, post-processed by filling holes smaller than 200 pixels (4-connectivity) and subsequently removing objects with an area smaller than 50 pixels (8-connectivity). (**c**) Segmented cells overlaid on the raw phase image. (**d**) Raw intensity image. (**e**) High intensity pixels (threshold 0.65) of the phase image were segmented. (**f**) Detached cell pixels/regions (threshold 0.35) were segmented from the intensity image. (**g**) Both masks were combined (OR-operation), holes smaller than 1000 pixels (4-connectivity) were filled, the regions were matched (AND-operation) with the phase mask (**b**) and regions smaller than 30 pixels (8-connectivity) were filtered out. (**h**) The remaining regions were used as seeds and dilated with a circular disk of 5 pixels. Next, these dilated regions were matched (AND-operation) with the detected cells in (**b**). The resulting detached cell mask was subtracted from the cell mask in (**b**) and regions smaller than 50 pixels (8-connectivity) were added to the detached cell mask (OR-operation). The final mask of detached cell regions was obtained. (**i**) Detached cell regions overlaid on the intensity image. (**j**) Segmented cells overlaid on the phase image, with attached and detached cell regions indicated by respectively a blue and a green mask.

A software tool was developed and implemented in MATLAB^©^ 2019a (MathWorks, MA, USA) to extract the novel feature, defined as the ratio of detached cell regions to the total number of cell pixels, from the reconstructed phase and intensity images. In Fig. 2, the segmentation procedure was visualised. The phase image was segmented using a random forest classifier, trained with the ‘Pixel classification’ workflow in Ilastik^21^. The standard feature variables for color/intensity, edge and texture were considered with a sigma value of 0.7, 1, 1.6, 3.5, 5 and 10. In total, 36 features were used for the classification. The classifier was trained on several images from different donors, ranging from attached to detached cells. The batch classification of all phase images was executed in Python using the “headless” mode of Ilastik. The subsequent steps were explained in more detail in Fig. 2. Moreover, the cell masks in Fig. 2B were used to extract the cell circularity, defined as $$Circularity= \frac{4*Area*\pi }{{Perimeter}^{2}}$$, and averaged over the cell population.

### Quantitative assessment

The detachment feature was quantitatively validated for three different culture times (after 3, 5 and 7 days in culture, using the non-diluted datasets). For Day 3, 401 × 401 pixel images were cropped around the centre of the reconstructed images, randomly selected over time for the different donors. For Day 5 and 7, the same procedure was followed, but 301 × 301 pixel images were used due to the higher cell densities. In total, each validation dataset consisted of 16 images. Using the brush tool in the Image Labeler app in MATLAB^©^ 2019a (MathWorks, MA, USA), the attached and detached cell regions were manually labelled for each phase image. Next, the percentage of detached cell regions was computed by dividing the number of detached pixels by the total number of labelled pixels (i.e. detached and attached). To validate the method, the absolute percentage error was computed according to formula (1):

1 $$Absolute \, error (\%)= {abs(Detached\, cell \%}_{man}-{Detached\, cell \%}_{auto})$$

With “man” and “auto” referring to the manual and automated extraction of the percentage of detached cell regions, respectively. For each culture time, the absolute percentage errors were represented as mean ± std.

### Harvesting time

For all conditions, the optimal time-point to inhibit the reaction was determined by applying a fixed threshold of 92.5%. This threshold was confirmed by visual inspection of the detached cell cultures at the respective inhibition times. Additionally, Supplementary Videos (4–7) were constructed to visualise the detachment response and highlight the optimal time-point for inhibition. In case the threshold was not reached within the period of 20 min, the final percentage of detached cell regions was indicated.

## Results

### Identification of a novel feature to describe cell detachment

**Figure 3:**
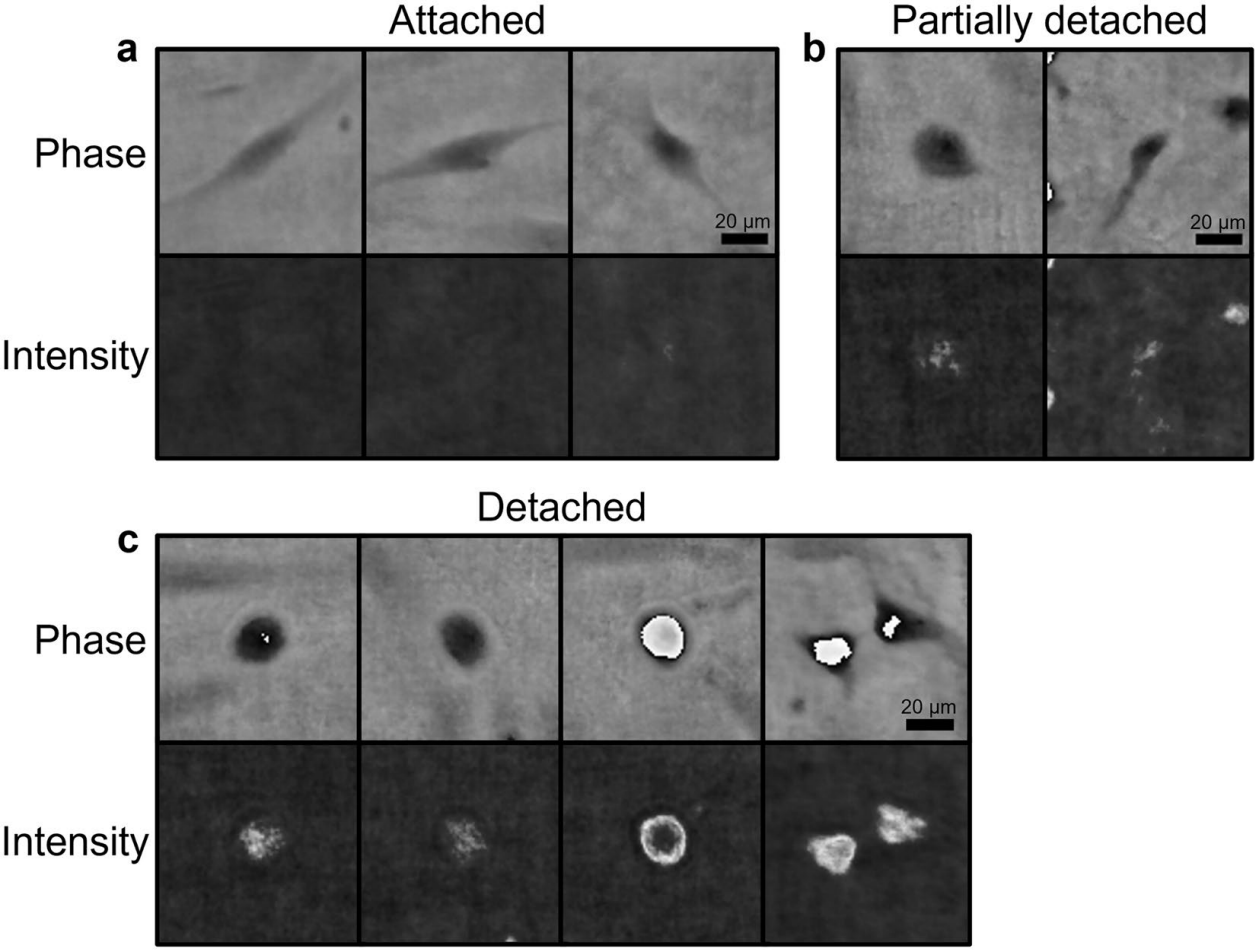
Visualisation of the novel cell detachment feature. Raw phase and intensity images of (**a**) attached, (**b**) partially detached and (**c**) detached cells.

**Figure 4:**
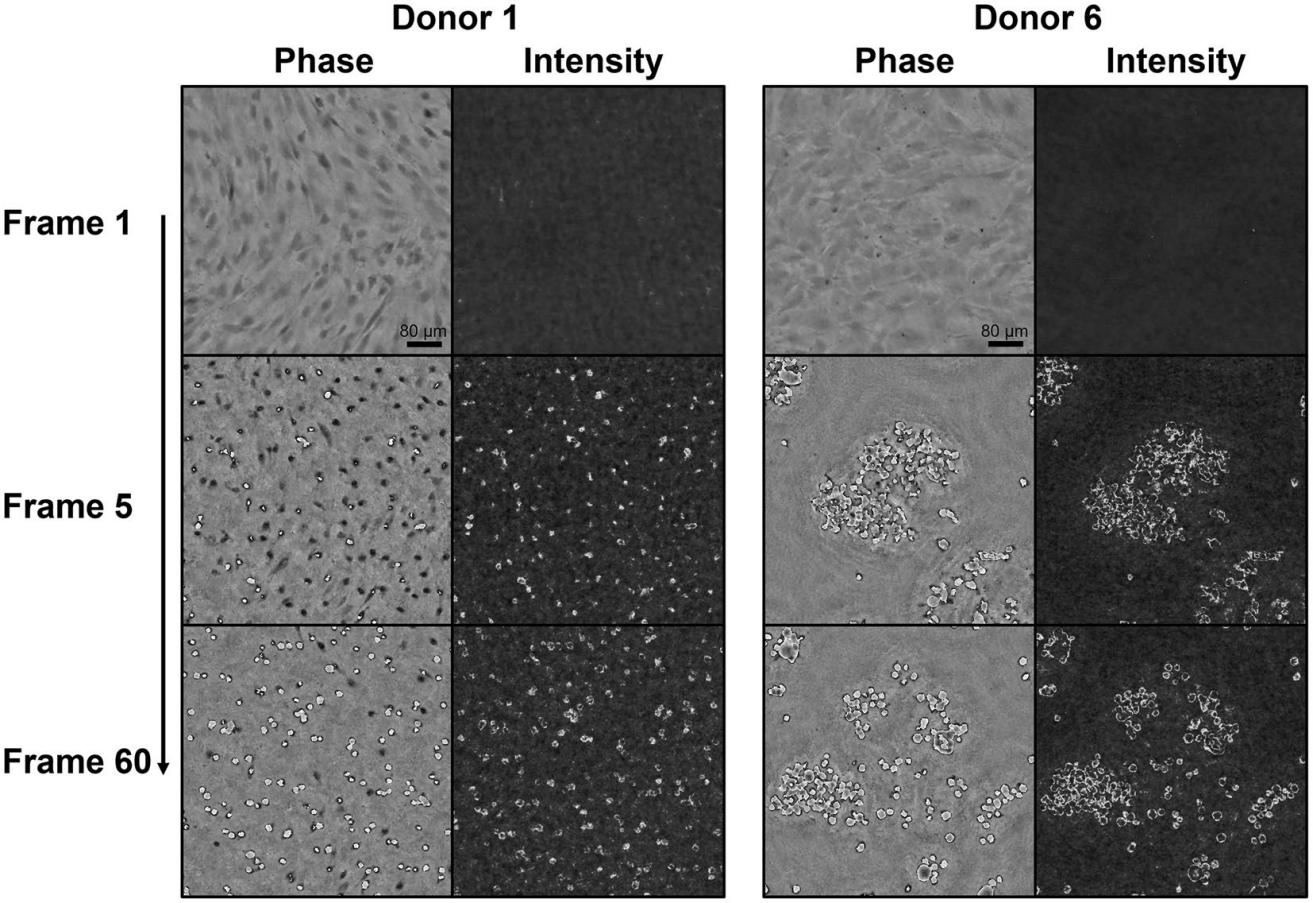
Visualisation of the detachment response for two different donors. The raw phase and intensity image were shown at frame 1, 5 and 60.

In this study, cell detachment was represented by a novel feature, derived from lens-free imaging data. In Fig. 3, several examples of attached, partially detached and detached cells are shown. Upon detachment, the hPDCs evolved from a long, stretched shape towards a circular shape, while their intensity initially decreased, followed by a bright halo artifact upon complete detachment from the substrate (i.e. floating). An alternative measure was developed through the optimisation of the LFI reconstruction parameters. Based on visual inspection, the influence of several parameters such as the reconstruction method, iteration count and IPR threshold on the reconstructed intensity images was evaluated (Supplementary Fig. S1). Since the 3L method did not return a clear visual distinction between attached and detached cells, the IPR reconstruction method was selected, with the optimal values for the IPR threshold and iteration count fixed at 90 and 10, respectively. Both a change in the IPR threshold and the iteration count decreased the correlation between bright regions in the intensity image and the visibly detached cells in the phase image. Increasing the IPR threshold from 90 to 105 resulted in noisy regions, while decreasing it to 80 caused an underestimation of the detached cell regions. For the iteration count, the opposite occurred. The focus was manually determined at the focal plane of the attached cells in the phase image. As a result, less noise was present in the intensity image while the bright cell regions corresponded better to the detached cells in the phase image. Using these parameter values, the appearance of bright pixel regions in the intensity images could be linked to specific characteristics of cell detachment in the phase image and therefore used as a measure for cell detachment. In Fig. 4, cell detachment was visualised for two donors with different detachment behaviour. Initially, all cells were attached to the substrate. Upon addition of the harvesting solution, the cells of both donors started to detach and detached cells appeared in the intensity images as bright regions, with considerably more clumping behaviour for donor 6. Towards the end of the harvesting assay, the majority of the cells were detached from the well plate. Based on the abovementioned observations, a novel feature was extracted (described in Fig. 2), defined as the percentage of detached cell regions.

### Quantitative assessment of the algorithm

**Figure 5:**
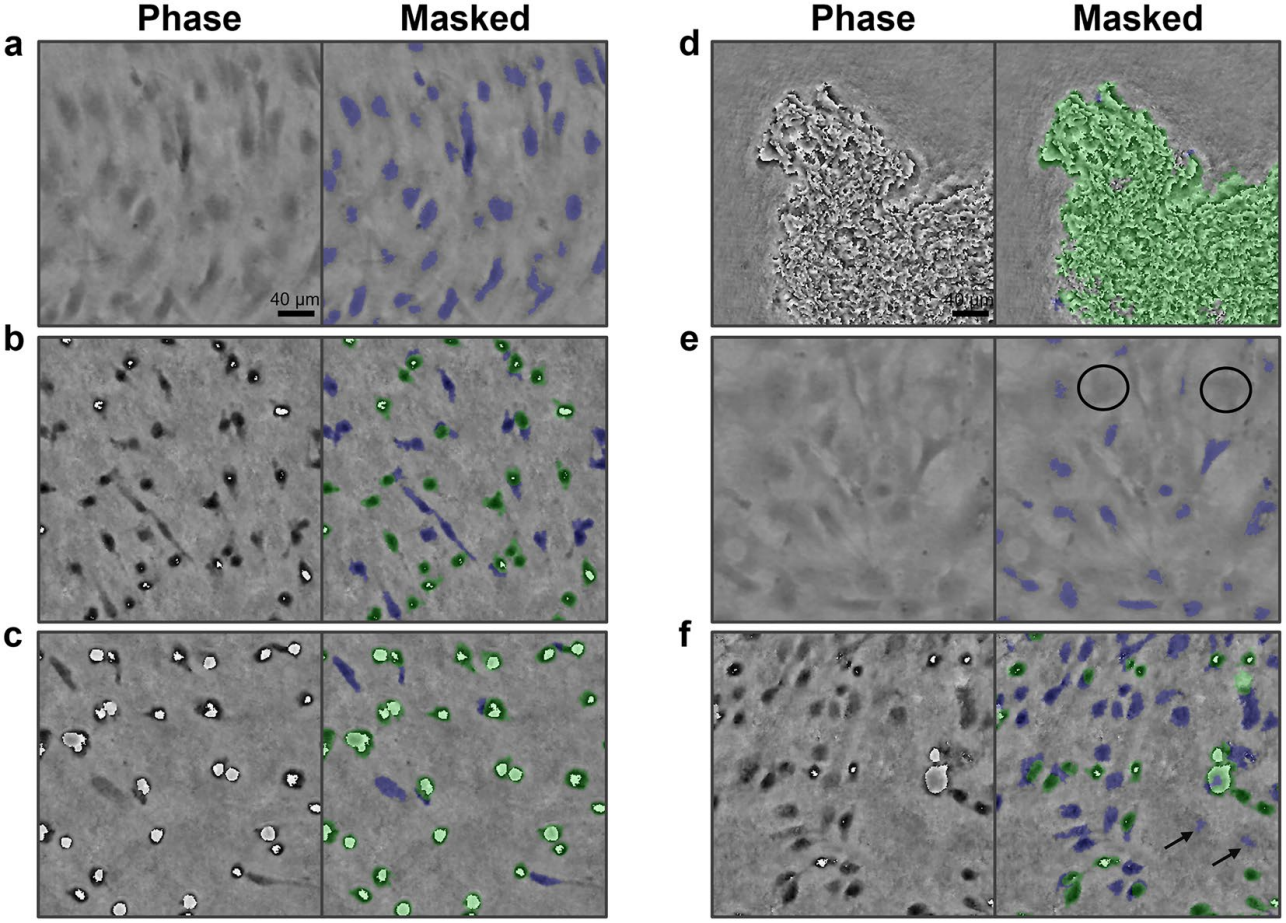
Qualitative validation of the developed algorithm for the detection of attached and detached cell regions. The validation was shown for 6 out of the 35 different datasets, at randomly selected timepoints, with: (**a**) donor 1 (day 7, no dilution, frame 1), (**b**) donor 3 (day 5, no dilution, frame 4), (**c**) donor 7 (day 7, no dilution, frame 55), (**d**) donor 6 (day 7, diluted, frame 10), (**e**) donor 6 (day 5, no dilution, frame 1) and (**f**) donor 5 (day 5, diluted, frame 15). The segmented pixel regions were overlaid on the raw phase image, with attached and detached cell regions displayed in blue and green, respectively. Examples of false negative and false positive regions were indicated by a black circle and arrow, respectively.

The methodology, developed to automatically extract the attached and detached cell regions for the phase image, was quantitatively and qualitatively validated on randomly selected images, representing different culture times (i.e. cell densities and matrix formation), donors and harvesting solutions (diluted or not). As shown in Table 1, average absolute error values ranging from 1.34 to 1.97% were obtained for the different culture times. Overall, the culture time did not have a large impact on the accuracy of our method, with similar error values obtained for medium density and confluent cultures and only slightly higher error values for overconfluent cultures. The quantitative results were also confirmed by Fig. 5, with most cells accurately detected by the algorithm. However, some cells were also undetected (black circle), usually at the start of the harvesting process where the attached cells exhibited lower contrast to the background. Additionally, a few false positive cell regions were also observed, indicated by an arrow in Fig. 5. Overall, the highlighted regions of attached (blue) and detached (green) cells matched well with the visual evaluation of cell detachment by an operator. The algorithm performed well for a wide range of cell densities and could also deal with clumping behaviour upon cell detachment. Additional examples are shown in Supplementary Videos 1–3.

**Table 1:** Quantitative validation of the developed algorithm for the extraction of the percentage of detached cell regions.

Culture time/cell density	Absolute error for percentage of detached cell regions (%)
Day 3	1.49% ± 1.25
Day 5	1.34% ± 1.19
Day 7	1.97% ± 1.45

The absolute errors are grouped per culture time (i.e. day 3, 5 and 7) and represented as mean ± std. Each validation dataset is composed of 16 images (n = 16).

### Automatic extraction of detachment profiles for individual cell donors

For optimal enzyme activity, the detachment response of the cells to the enzymatic solution was monitored at 37 °C in a standard cell culture incubator. With the LFI set-up, a minimum interval of 20 s was achieved, confirmed by the temperature measurements in Supplementary Figure S3. Over a period of up to 5 h, only a small heating effect was observed with respect to the equilibrium phase, while no heating of the media was observed within the first 20 min. In case the imaging sensor was not turned off between image acquisitions, the medium and local air temperature increased up to 40.3 °C and 41.9 °C, respectively.

**Figure 6:**
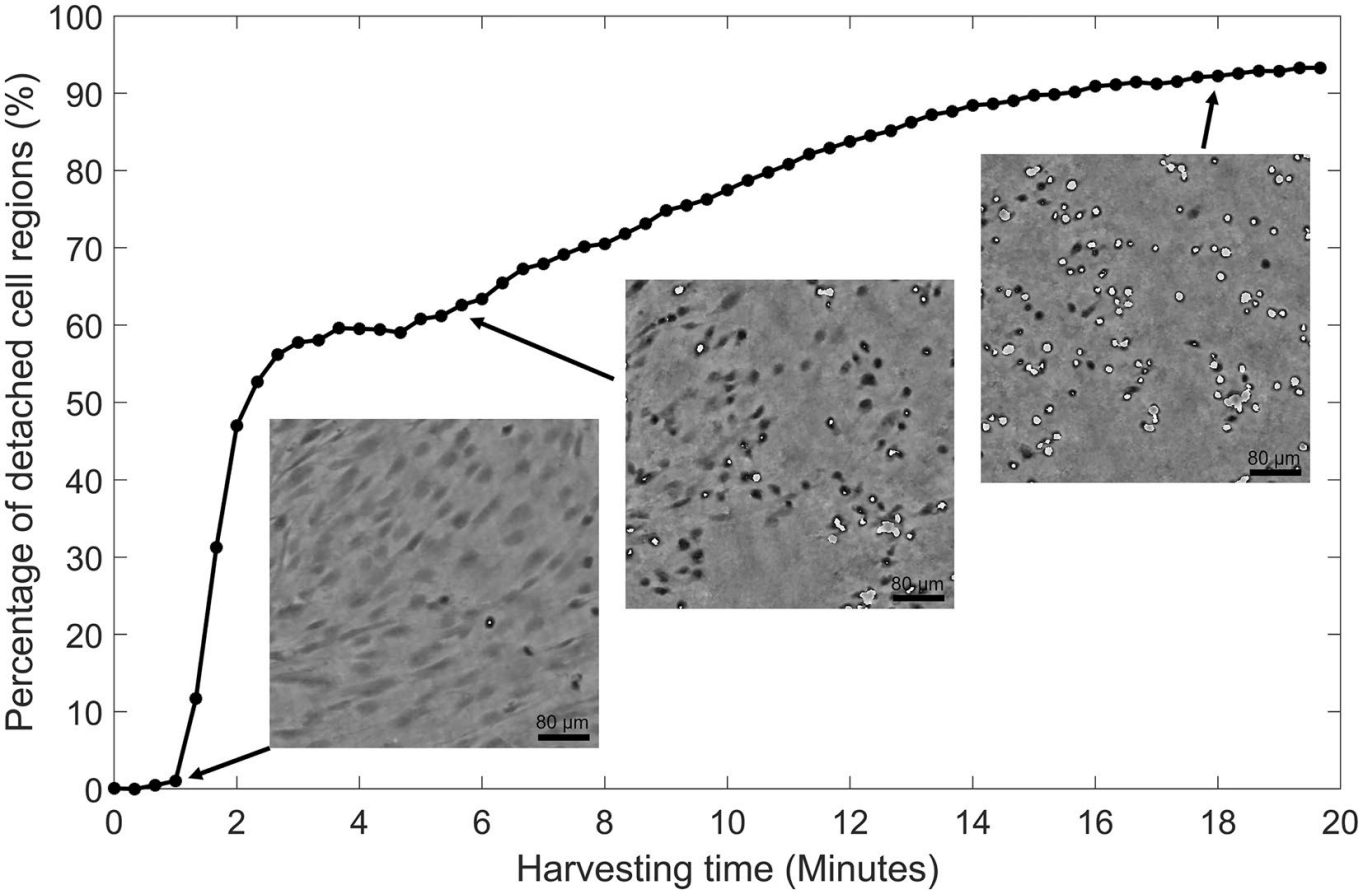
Illustration of the cell detachment response. The percentage of detached cell regions with respect to the total cell area, with the corresponding phase images at frame 4, 18 and 55.

**Figure 7:**
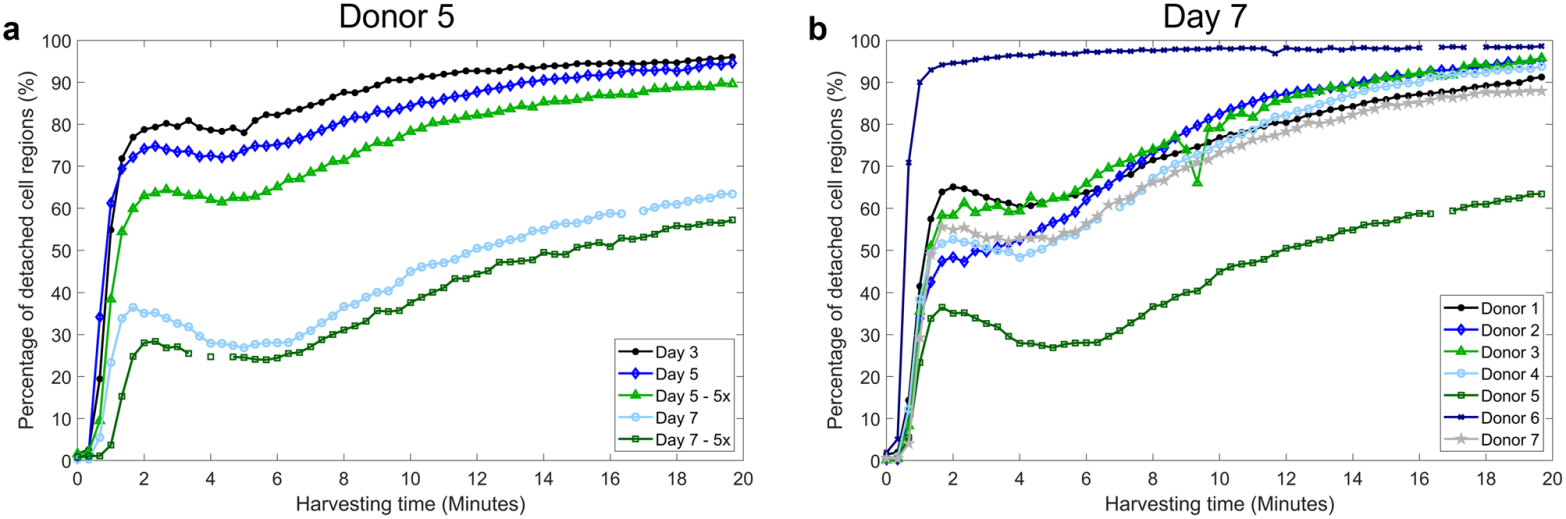
Cell detachment over time. (**a**) Detachment response of donor HP297 for the different conditions. (**b**) Detachment response of the different donors after 7 days in culture (enzymatic solution not diluted).

An example of the cell detachment response with automated monitoring is shown in Fig. 6, while in Fig. 7 the percentage of detached cell regions was plotted in function of the harvesting time and a visual comparison was shown for different conditions (i.e. culture time and dilution) and different donors. For most conditions, a strong initial detachment phase was observed, followed by a short stationary or re-attachment phase and a slower detachment response until a plateau was reached. The number of cells that initially detached largely depended on the donor, culture time (i.e. cell density and matrix formation) and the dilution of the enzymatic solution. For donor 5, the detachment rate decreased with culture time, while the effect of the dilution factor was less pronounced (Fig. 7A). In Fig. 7B, five out of seven donors exhibited a similar detachment response after 7 days in culture, while donor 5 and 6 respectively detached much slower or faster than the average donor. Similar trends were observed for day 5 and 7 with the diluted harvesting solution, but slightly shifted for the other conditions (data not shown).

### Automatic determination of the inhibition time

**Figure 8:**
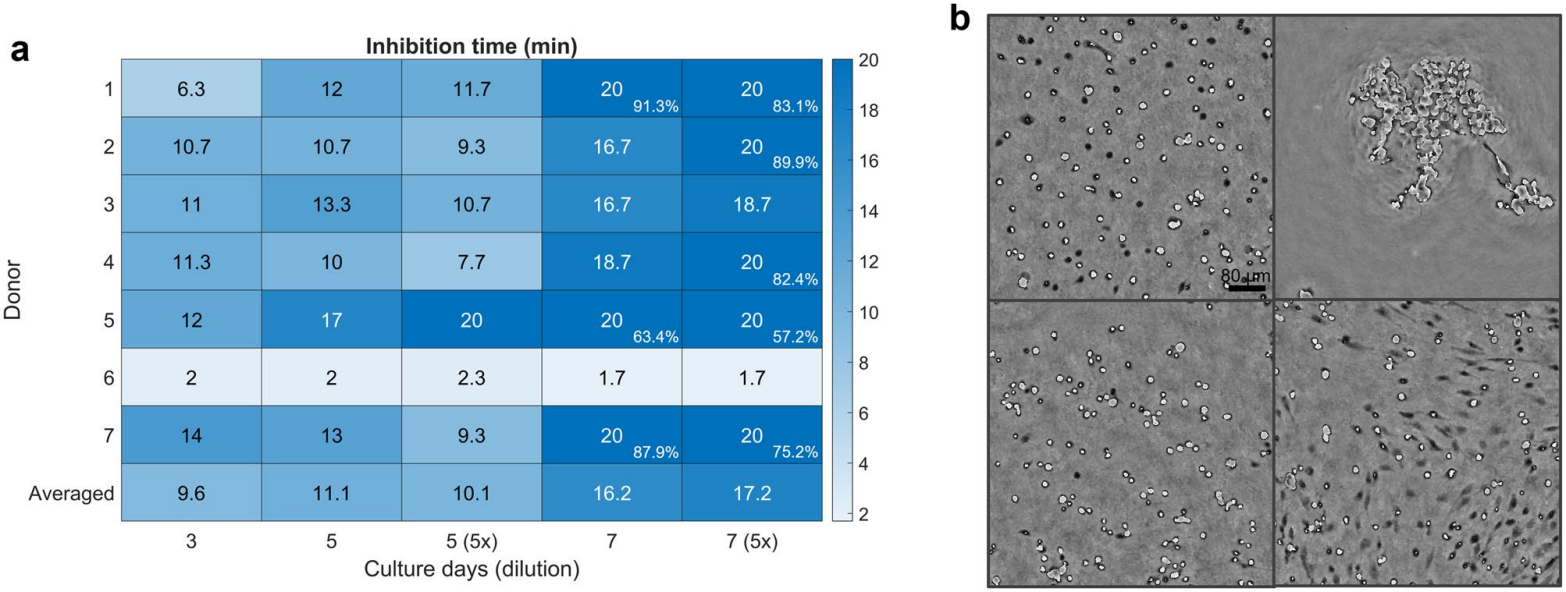
Inhibition times extracted using the threshold approach. (**a**) Heat map of the inhibition time for the different conditions. In case the threshold was not reached within the 20-min harvesting period, the final percentage of detached cells was indicated in the bottom-right corner. (**b**) Four examples of phase images at their corresponding inhibition times. Top left: donor 2 (day 5, no dilution). Top right: donor 6 (day 5, diluted). Bottom left: donor 3 (day 7, diluted). Bottom right: donor 5 (day 7, diluted).

The optimal time to inhibit the enzymatic reaction was automatically determined. In Fig. 8, a heat map was shown to display the inhibition time for each condition. The average harvesting time was roughly 10–11 min for medium (day 3) to high (day 5) cell densities, while it increased up to 16–17 min for overconfluent cell cultures. The latter is still an underestimation of the inhibition time, since in more than 50% of the conditions, the threshold of 92.5% was not reached within the 20-min harvesting period. For the culture time, on average, only a small increase of 1.6 min was observed from day 3 to day 5, while a larger increase of approximately 5 min was noticed from day 5 to 7. The dilution factor of the TrypLE solution, on the other hand, did not seem to have a large impact on the inhibition time. On average, a decrease of 1.2 min and an increase of 0.8 min was observed for the day 5 and day 7 cultures, respectively. However, 4 out of 7 donors reached the harvesting threshold for the standard solution on day 7, while only 2 out of 7 for the diluted solution, and with lower cell yield percentages after 20 min (donor 1, 5 and 7). On a donor level, donors 1–4 and 7 roughly exhibited the same trend for their inhibition times, while donor 5 and 6 clearly showed a distinct behaviour. For HP297, large inhibition times were reported, increasing with culture time and dilution, while HP449 detached very abruptly for all conditions.

In order to verify the chosen threshold, phase images of the detaching cells were checked at the proposed inhibition times (Fig. 8B). Overall, a fixed threshold of 92.5% achieved consistent detachment results across the different conditions, with most of the cells detached from the substrate. In Supplementary Videos 4–7, more examples of the inhibition time were shown.

## Discussion

Although enzymatic harvesting has been widely used for adherent cell cultures, it is still lacking a standardised and reproducible inhibition step, necessary for the cultivation of consistent cell batches ^22^. On-line monitoring systems can provide an objective evaluation of the cell detachment response, thereby assisting in the optimisation and automation of cell harvesting processes. In order to meet the current needs of the field, scalable set-ups that apply to a wide range of conditions are required.

Traditionally, cell circularity is used to monitor the detachment response of adherent cells^11,18^, while template matching also has been applied for cell attachment^18^. Recently, the automated extraction of cell parameters such as the cell circularity has been simplified by the development of several advanced monitoring systems^12,13^. However, accurate segmentation of individual cells is still challenging, especially for highly confluent cell cultures^23^. Moreover, advanced models such as deep learning frameworks^12,13^ are often tailored to specific conditions and lack robust performance for high density to overconfluent cell cultures. For our lens-free images, the distinction between adherent single cells also decreased for high cell densities, while occasional cell clumping behaviour upon detachment made it sometimes impossible to delineate individual cells (Supplementary Fig. S2). In order to avoid these challenges, the novel feature does not rely on the cells their morphological characteristics, but rather on the appearance of detached cells in the intensity image upon detachment. As shown in Table 1, this resulted in accurate results over a wide range of densities (up to overconfluency), while algorithms relying on single cell segmentation often are less accurate^13^, especially when the cell culture is becoming (over) confluent^12^. This is also illustrated in Supplementary Fig. S2, where the novel feature showed robust behaviour for different cell densities and donors, while the correlation with the circularity significantly decreased for high densities as a result of touching cells. However, it has to be noted that the segmentation procedure was not optimised for the detection of single cells, and therefore only serves as an example of inappropriate single cell segmentation at high density. Moreover, the dilation of the detached cell masks (Fig. 2) improved the consistency of the feature while it decreased the sensitivity towards small deviations in the reconstruction depth. In general, it is believed that the novel feature is inherently more generic than the circularity measure since it tries to directly quantify the adherence of the cells to the culture vessel. Moreover, cell circularity is not always a good representation for the detachment state of the cell, as shown in Supplementary Fig. 4B, while for template matching it can be challenging to find a representative library of templates to detect detaching cells (Supplementary Fig. 4A). The novel feature is unique to LFI, and a similar approach on conventional images would be challenging. Opposite to our lens-free phase images, phase-contrast images capture the whole cell region. This can result in a significant underestimation of the percentage of detached cell regions, especially when only a small number of cells are detached. In addition, the contrast of the cells in conventional bright-field images increases upon detachment, but not as pronounced as for the LFI intensity image. Next to the novel feature, LFI technology also offers other advantages with respect to conventional microscopy. It is a cost-effective and compact imaging technique and therefore has great potential for parallelisation^24^ and close integration with culture vessels such as microfluidic set-ups^25^. However, it is also characterised by a lower resolution and is more sensitive to condensation and other disturbances (e.g. vibrations). Therefore, it has to be well evaluated whether it is more favourable to use a LFI system or conventional set-up for a certain application.

In cell harvesting applications, the detachment rate of the cells can be relatively high, especially at the start of the harvesting process. Therefore, a short time interval of 20 s was preferred to continuously monitor the culture. As a result of the LFI electronics, a small increase in air and medium temperature was already observed in equilibrium (i.e. no imaging), while this increased further during the measurements (heating of the sensor). However, no adverse effects on the cell culture were expected for this temperature and time period^26–28^, while the temperature increase will also be lower for the 6-well plate set-up due to the increased distance of the wells to the imaging sensor and LFI electronics. In case there would still be a negative impact on the culture, the temperature of the incubator could be slightly reduced during cell harvesting. Monitoring the detachment response of the cells with a high imaging frequency provided some interesting insights into their detachment dynamics. For most donors, two different detachment rates were observed within the 20-min time frame, which could be a result of the heterogenic nature of hPDC cultures^29^. Cell types are characterised by their adhesion strength^30^ and therefore release time. In turn, this could also explain the differences observed between individual donors, with donor 5 and 6 seemingly composed of mainly slow and fast detaching cells, respectively. The stationary/re-attachment phase, on the other hand, was an unexpected result and to our knowledge reported for the first time. Since it was not captured by the circularity feature (Supplementary Fig. S2), previous studies have likely missed this phenomenon.

Next to monitoring the cell detachment response, an accurate determination of the inhibition time is required. In Viazzi et al*.*^11^*,* cell circularity was used to characterise the cell detachment response to different harvesting solutions. The response was described by a first-order transfer function (TF) model, enabling the extraction and on-line prediction of the inhibition time. However, this approach is not applicable to conditions that deviate from a first-order model, while the averaged circularity does not provide an indication on the cell yield. In contrast, our novel feature is a direct representation of the detached state of the cells, quantified as the percentage of detached cell regions. Therefore, it is a measure for the cell yield, and the optimal inhibition time can be determined by choosing the desired threshold for the percentage of detached cell regions. Since this approach does not require the use of a generalised model, it will be more generic across cell types and conditions. In order to automate the inhibition step, the threshold method could be used to initialise the harvesting procedure. Future research in an on-line setting will allow us to validate the threshold through manual counting of the cell yield.

Using the current threshold, average inhibition times around 10 min were obtained for mid to high cell densities (day 3/5). These times correspond well to our general lab protocol, in which cell cultures are grown up to 80–90% confluency and subsequently detached from the substrate with TrypLE solution for approximately 10 min. It confirms the efficacy of the algorithm and chosen threshold for real-life cell harvesting applications. Moreover, the large inter-donor differences that were observed (especially for donor 6) reaffirmed the need for data-based monitoring technologies, enabling individualised enzyme exposure times. Since trypsin or any other proteolytic agent can have severe adverse effects on the cell population^5–8^, these set-ups will be important to control and minimize the exposure time to the enzymatic solution. Moreover, interesting results were obtained with respect to the culture time and dilution factor. For most donors, the detachment rate decreased significantly when going from high cell density (day 5) to overconfluency (day 7), likely a result of the dense cell stacking and matrix deposition. The dilution factor of 5, on the other hand, only had a small impact on the inhibition time for overconfluent cell cultures. However, lower final percentages of detached cells were obtained for the diluted solution, which indicates that the difference in average inhibition time is underestimated. Preferably, enzymatic solutions are diluted without compromising the harvesting time, reducing the impact of the harvesting solution on the cells. It has to be noted that no replicates were included in the experiment and therefore no biological conclusions could be drawn from the existing data. However, the study showed the potential of the novel feature and data-based approaches for automated decision-making in cell harvesting. In addition, the set-up can be used to further optimise the harvesting process by investigating the effect of different parameters on the inhibition time. We believe that the developed method can also be generalised to other LFI systems, given that a similar reconstruction algorithm is used for the intensity images.

## Conclusion

A novel feature, derived from lens-free images, was used to monitor the detachment response of adherent cell cultures. The feature showed robust behaviour for different cell donors, densities and harvesting solutions. Moreover, it is an interpretable feature which is potentially more generic than other existing features. Next to cell harvesting, the feature could also have important implications for monitoring cell division and cell death, since these processes are also characterised by a (partial) detachment of the cells from the culture vessel. In a next step, the developed methodology could be deployed in an on-line setting to evaluate the actual cell yield after cell harvesting. Moreover, it can be validated for other set-ups such as cell culture flasks or small-scale bioreactors. Together with the inexpensive and compact LFI design, it can enable the implementation of automated harvesting strategies on a large scale, thereby minimizing the risk of irreversible cell damage while maximizing the cell yield.

## Supplementary Information

Supplementary Information.

Supplementary Video 1.

Supplementary Video 2.

Supplementary Video 3.

Supplementary Video 4.

Supplementary Video 5.

Supplementary Video 6.

Supplementary Video 7.

## Data Availability

The datasets generated during and/or analysed during the current study are available from the corresponding author on reasonable request.
