# Supplementary material for: A novel feature for monitoring the enzymatic harvesting process of adherent cell cultures based on lens-free imaging
Source: Sci Rep. 2022 Dec 23;12:22202. doi: 10.1038/s41598-022-22561-x (PMC9789138; doi:10.1038/s41598-022-22561-x)
Supplement: Supplementary file 1 — Supplementary Information. [file 41598_2022_22561_MOESM1_ESM.pdf]

Supplementary Material

A novel feature for monitoring the enzymatic harvesting process of adherent cell cultures based on lens-free imaging

T. Deckers[1,2,3], J.-M. Aerts[1,3], V. Bloemen[2,3,*]

Affiliations
[1]M3-BIORES, KU Leuven, Leuven, Belgium. [2]Surface and Interface Engineered Materials (SIEM), Campus Group T, KU Leuven, Leuven, Belgium. [3]Prometheus, Division of Skeletal Tissue Engineering Leuven, KU Leuven, Leuven, Belgium.

Contact information: veerle.bloemen@kuleuven.be

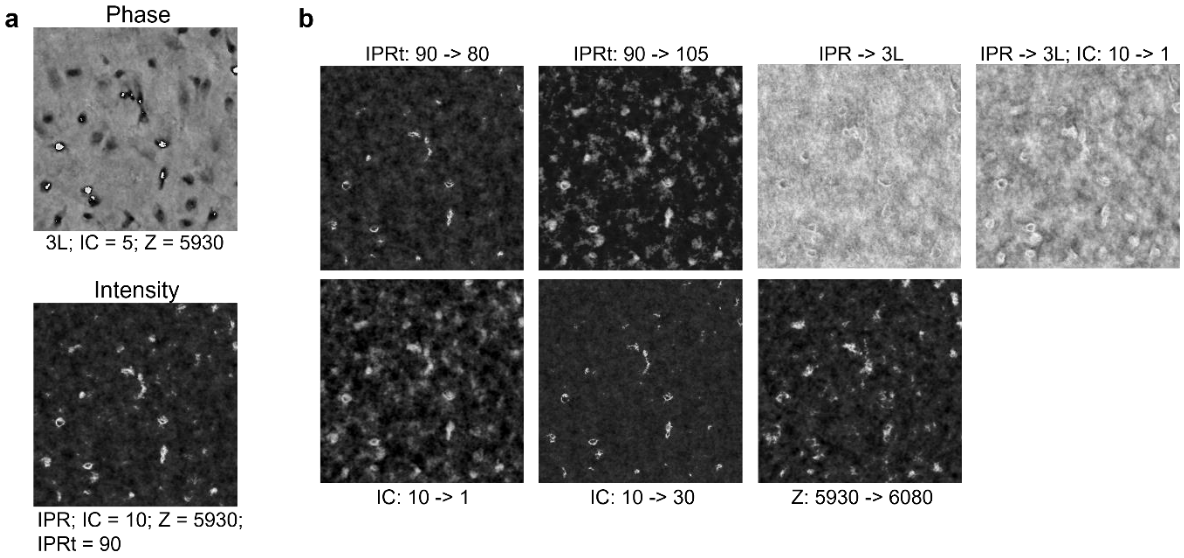

Supplementary Figure S1. Effect of different parameters on the reconstruction of the intensity image. (**a**) Phase and intensity image reconstructed with the optimal parameters. (**b**) The values of different parameters were varied for the intensity image. Parameters: reconstruction method (3L or IPR), iteration count (IC), focal plane (Z) and IPR threshold (IPRt).

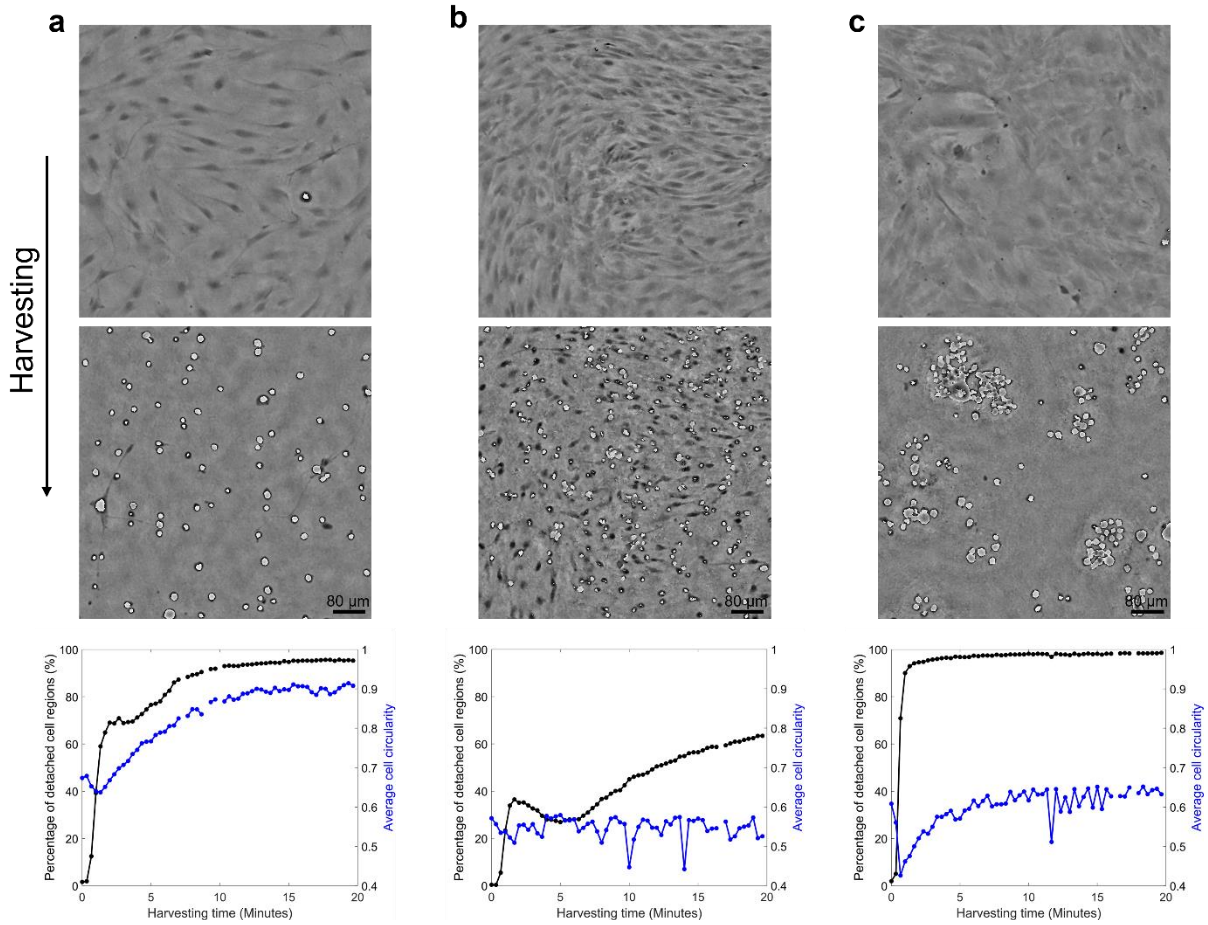

Supplementary Figure S2. Comparison between the novel and the circularity feature. The detachment response was displayed (phase image) along with the novel (black) and circularity (blue) feature for three different conditions: (**a**) donor 2 (day 3, no dilution), (**b**) donor 5 (day 7, no dilution) and (**c**) donor 6 (day7, no dilution).

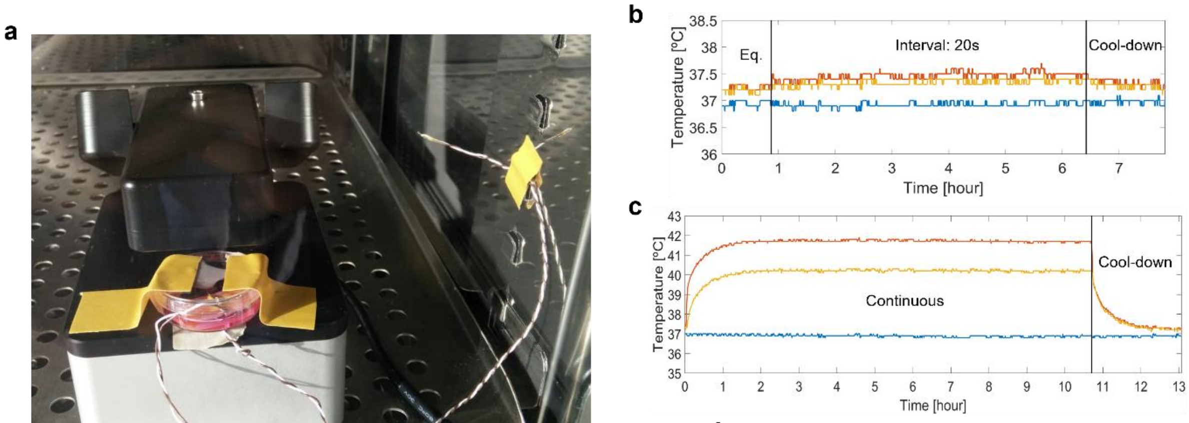

d

Sensor/Mode	Equilibrium	Interval:20s	Continuous
Reference	37.0	37.0	37.0
Air	37.3	37.7	41.9
Medium	37.3	37.5	40.3

Supplementary Figure S3. Monitoring the temperature response during image acquisition for a standard culture dish. (a) A petri-dish was used as a set-up for the temperature measurements**.** Three thermocouples were used to perform the measurements, coupled to a DaqPro to record the data. The first thermocouple was mounted in the right-bottom corner of the incubator (sensor: reference) and was used as a reference. The second couple was glued to the bottom of the petridish (sensor: air) while the third one was glued inside the petri-dish (sensor: medium). The petridish was filled with a small amount of medium and fixed to the LFI stage. The petri-dish was aligned with the stage such that both thermocouples were visible in the center of the field of view. Prior to the measurements, the incubator was kept closed for a few hours for temperature stabilization. The temperature was recorded while the LFI was in **(b)** time-lapse mode (for 5 and ½ hours) with an interval of 20 seconds, i.e. the small interval for which the LFI/image sensor is still switching on/off in between image recordings or **(c)** continuous mode (for up to 12 hours). Both temperature measurements were recorded with a time interval of 10 seconds (and rounded to 1 decimal) and were preceeded by an equilibrium phase (temperature measurements while LFI is off). **(d)** Maximum temperature for a certain sensor and mode.

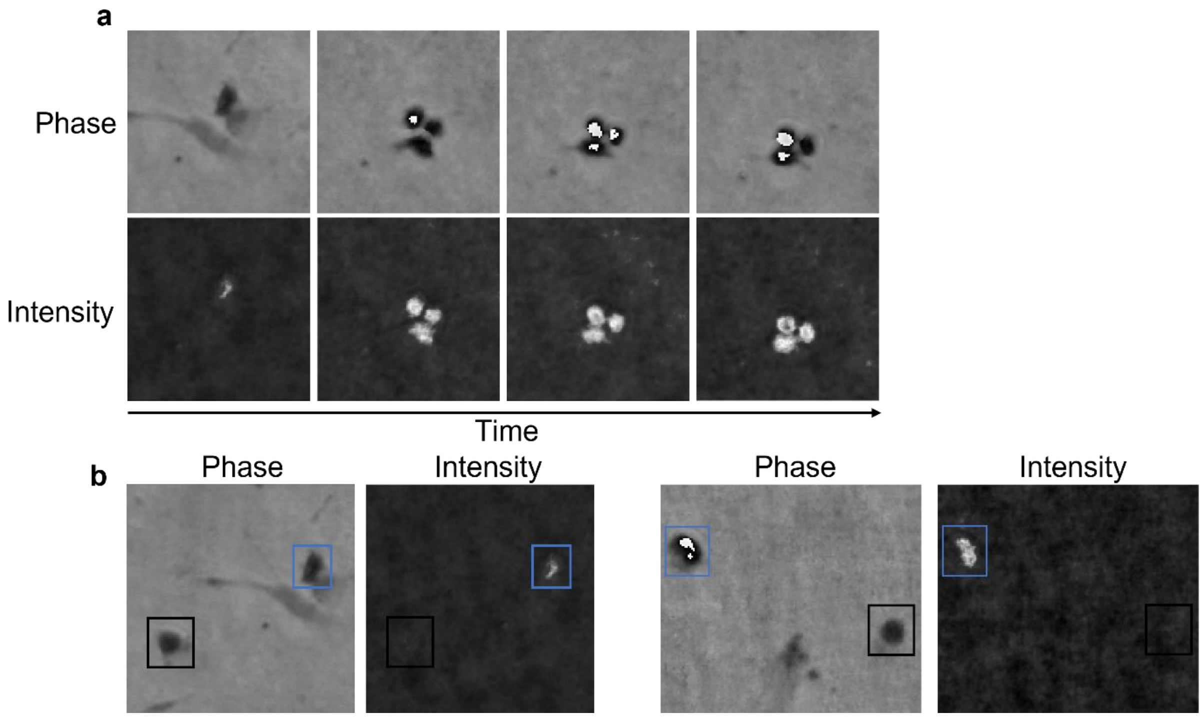

Supplementary Figure S4. Visual comparison of different features for representing cell detachment. (a) Detachment of cells over time, with both the phase and intensity images shown. Detached cells can adopt different shapes (from oval to circular) and intensity distributions (from low to high intensity), resulting in a broad range of appearances. **(b)** Cell examples for which roundness/circularity is not representative of cell detachment. Black squares indicate round cells which are still attached, while blue squares are oval-shaped cells which are detaching or detached, opposite to the expected appearance upon detachment.

Supplementary Video 1. Qualitative validation of the feature extraction. Visualisation of the detachment response of donor 3 (day 5, no dilution) over a period of 20 minutes. On the right image, the raw phase image was overlaid with the attached (blue) and detached (green) cell masks.

Supplementary Video 2. Qualitative validation of the feature extraction. Visualisation of the detachment response of donor 1 (day 7, no dilution) over a period of 20 minutes. On the right image, the raw phase image was overlaid with the attached (blue) and detached (green) cell masks.

Supplementary Video 3. Qualitative validation of the feature extraction. Visualisation of the detachment response of donor 5 (day 7, diluted) over a period of 20 minutes. On the right image, the raw phase image was overlaid with the attached (blue) and detached (green) cell masks.

Supplementary Video 4. Visual validation of the inhibition time. Visualisation of the detachment response of donor 2 (day 3, no dilution). When the threshold of 92.5% (indicated in green) was reached, the image titles turned from black to green.

Supplementary Video 5. Visual validation of the inhibition time. Visualisation of the detachment response of donor 4 (day 5, no dilution). When the threshold of 92.5% (indicated in green) was reached, the image titles turned from black to green.

Supplementary Video 6. Visual validation of the inhibition time. Visualisation of the detachment response of donor 6 (day 7, no dilution). When the threshold of 92.5% (indicated in green) was reached, the image titles turned from black to green.

Supplementary Video 7. Visual validation of the inhibition time. Visualisation of the detachment response of donor 5 (day 7, dilution). When the threshold of 92.5% (indicated in green) was reached, the image titles turned from black to green.
